# The mechanism of oxytocin and its receptors in regulating cells in bone metabolism

**DOI:** 10.3389/fphar.2023.1171732

**Published:** 2023-05-09

**Authors:** Liu Feixiang, Feng Yanchen, Li Xiang, Zhang Yunke, Miao Jinxin, Wang Jianru, Lin Zixuan

**Affiliations:** ^1^ The First Affiliated Hospital of Henan University of Chinese Medicine, Zhengzhou, Henan, China; ^2^ Traditional Chinese Medicine (Zhong Jing) School, Henan University of Chinese Medicine, Zhengzhou, Henan, China; ^3^ State Key Laboratory for Diagnosis and Treatment of Infectious Diseases, National Clinical Research Center for Infectious Diseases, Collaborative Innovation Center for Diagnosis and Treatment of Infectious Diseases, The First Affiliated Hospital, Zhejiang University School of Medicine, Hangzhou, China; ^4^ Research Units of Infectious Disease and Microecology, Chinese Academy of Medical Sciences, Hangzhou, China; ^5^ School of Rehabilitation Medicine, Henan University of Chinese Medicine, Zhengzhou, China; ^6^ Research and Experiment Center, Henan University of Chinese Medicine, Zhengzhou, China

**Keywords:** immune inflammation, epigenetics, oxytocin, oxytocin receptor, osteoporosis, estrogen, bone marrow mesenchymal stem cells, osteoblasts

## Abstract

Oxytocin (OT) is a neuropeptide known to affect social behavior and cognition. The epigenetic modification of the oxytocin receptor (OTR) via DNA methylation stimulates parturition and breast milk secretion and inhibits craniopharyngioma, breast cancer, and ovarian cancer growth significantly as well as directly regulates bone metabolism in their peripheral form rather than the central form. OT and OTR can be expressed on bone marrow mesenchymal stem cells (BMSCs), osteoblasts (OB), osteoclasts (OC), osteocytes, chondrocytes, and adipocytes. OB can synthesize OT under the stimulation of estrogen as a paracrine–autocrine regulator for bone formation. OT/OTR, estrogen, and OB form a feed-forward loop through estrogen mediation. The osteoclastogenesis inhibitory factor (OPG)/receptor activator of the nuclear factor kappa-B ligand (RANKL) signaling pathway is crucially required for OT and OTR to exert anti-osteoporosis effect. Downregulating the expression of bone resorption markers and upregulating the expression of the bone morphogenetic protein, OT could increase BMSC activity and promote OB differentiation instead of adipocytes. It could also stimulate the mineralization of OB by motivating OTR translocation into the OB nucleus. Moreover, by inducing intracytoplasmic Ca^2+^ release and nitric oxide synthesis, OT could regulate the OPG/RANKL ratio in OB and exert a bidirectional regulatory effect on OC. Furthermore, OT could increase the activity of osteocytes and chondrocytes, which helps increase bone mass and improve bone microstructure. This paper reviews recent studies on the role of OT and OTR in regulating cells in bone metabolism as a reference for their clinical use and research based on their reliable anti-osteoporosis effects.

## 1 Introduction

Oxytocin (OT), which was discovered in 1906, is a neuropeptide with a relative molecular mass of 1,007 (Da) and is composed of cysteine–tyrosine–isoleucine–glutamine–asparagine–cysteine–proline–leucine–glycine (Froemke and Young, 2021; Camerino C, 2023). It is primarily synthesized in the supraoptic and paraventricular hypothalamic nuclei, transported to the posterior pituitary for storage, and released into the bloodstream in response to appropriate stimulation (Tunheim et al., 2023). Additionally, some peripheral tissues and organs can also synthesize and secrete OT, such as the uterus, ovary, villi, placenta, testis, adrenal gland, thymus, pancreas, and heart (Kerem and Lawson, 2021). Oxytocin receptors (OTRs) are required to mediate the physiological function of OT, which belongs to the rhodopsin-like (class A/1) G protein-coupled receptor family. It responds to the neurohypophysial hormone OT to stimulate lactation and social behaviors and is also expressed within a variety of cells (Waltenspühl et al., 2020).

OT has the functions of promoting contractions of uterine smooth muscle in delivery and aiding in lactation after childbirth. OT pathway genes confer individual differences in social cognition and personality in humans, which could be explained by the presence of intermediary, epigenetic, variables that exist between the genotype and phenotype (Haas et al., 2018). Meanwhile, as an evolutionary ancient and widely distributed signaling system, the OT/OTR signaling system could be utilized by many cancer types, including neuroblastoma and glioblastoma, small-cell lung carcinoma, endometrium adenocarcinoma, and ovarian carcinoma (Cassoni et al., 1998; Morita et al., 2004; Pequeux et al., 2004; Busnelli et al., 2010). It is believed that OT could reduce the proliferation and migration of three ovarian cancer cell lines, induce apoptosis and autophagy, and partially reverse the effects of cortisol (Mankarious et al., 2016). With the in-depth study of the pituitary–bone axis, more sources of evidence prove that OT and OTR play an essential role in regulating bone metabolism (Kim et al., 2022; Winterton et al., 2022). By reviewing the relationship progress between OT/OTR and bone marrow mesenchymal stem cells (BMSCs), osteoblasts (OB), osteoclasts (OC), osteocytes, adipocytes, and chondrocytes, the mechanism of OT/OTR in regulating bone metabolism is clarified, which can provide certain reference values to clinical application with their anti-osteoporosis effects.

## 2 Central OT and peripheral OT

Central OT (COT) is synthesized in the hypothalamus and released into circulation by the neurohypophysis, which plays a significant role in milk excretion and uterine contraction at childbearing age (Ferreira et al., 2019). Although it can increase trust, motivate social participation and group preference, and relieve social stress response as well as has anti-inflammation effects, there is no synthetic metabolic impact on bones (Breuil et al., 2011; Hood et al., 2020; Sindermann et al., 2020; Festante et al., 2021). The evidence suggests that the injection of OT into the lateral ventricle has no effect on OB and OC formation as well as sero-bone conversion markers (Tamma et al., 2009).

Peripheral OT (POT) is produced in adipocytes, OB, uterus, ovary, testis, and other tissues that participates in breastfeeding and delivery and plays a direct role in bone homeostasis. Clinical studies have shown that the OT plasma level in postmenopausal women with postmenopausal osteoporosis (PMOP) is significantly lower than that in non-PMOP patients. The low level of OT plasma could further cause bone mass loss in women with severe PMOP (Breuil et al., 2011; Zofková and Matucha, 2014). The histomorphometry and micro-computed tomography analysis revealed that volumes of femur’s vertebral body and trabecula were significantly reduced for OT and OTR knockout mice. However, intraperitoneal injection of OT could effectively reverse the bone defect caused by decreased OB differentiation (Tamma et al., 2009). Similarly, intraperitoneal injection of OT into ovariectomized rats could significantly reduce the decrease in OB, the increase in OC, the reduction in the serum ratio of osteoprotegerin (OPG)/receptor activator of nuclear factor kappa-B ligand (RANKL), and the increase in bone formation transition markers (Ikebuchi et al., 2018). Additionally, the OT serum level in PMOP patients was significantly correlated with the severity of OP and the decline in bone mineral density rather than other pituitary hormones, indicating that the anabolism effect of OT on bones may be primarily related to the direct and peripheral actions of OB (Breuil et al., 2011).

These findings demonstrate that, as opposed to indirectly mediating bone metabolism via the central nervous system, the direct effect of OT on bones is primarily connected to the peripheral action of OT.

## 3 POT and cells regulating bone metabolism

### 3.1 Bone marrow mesenchymal stem cells

BMSCs are bone marrow stromal stem cells that could differentiate into OB, OC, adipocytes, chondrocytes, and myoblasts (Uzieliene et al., 2019; Huang et al., 2021). Fallahnezhad et al. (2018) have shown that POT could increase the potential of BMSCs to differentiate into OB and the cell survival of BMSCs from ovariectomized rats. The BMSC mechanism may be related to the upregulation of TGF, IGF-1, serum alkaline phosphatase (ALP), and OPG and the downregulation of runt-related transcription factor 2 (RUNX2) and osteocalcin. Furthermore, Santos et al. (2018) have shown that the combination of POT and osteogenic inducer could remarkably increase the content of POT and OTR in BMSCs for adults and aged female rats, promote the proliferation and osteogenic differentiation of BMSCs, and increase the activity of ALP and expression of bone morphogenetic protein-2 (BMP-2), osteopontin (OPN), and osteocalcin.

In conclusion, these studies demonstrate that POT could stimulate BMSC proliferation, enhance their activity, and induce OB differentiation and mineralization, which may be the key to boosting bone mineral density.

### 3.2 Osteoblasts

OB originate from BMSCs and are closely related to new bone formation, which are mainly responsible for the synthesis and secretion of bone matrix and the mineralization of bone matrix, thus playing a vital role in bone formation (Li et al., 2021). POT is produced by OB in the bone marrow and acts as a paracrine–autocrine regulator of bone formation (Elabd et al., 2008; Ein-Dor et al., 2018). OTR is also highly expressed in OB. The direct and dominant effect of OT on bone homeostasis is mainly achieved by stimulating the formation of OB and bidirectionally regulating the formation of OC. Studies have shown that the knockout of POT and OTR genes of OB in mice displayed decreased mineralization activity, and the major genes representing OB differentiation, such as osteocalcin, Runx2, and OPN, were considerably downregulated (Colaianni et al., 2014). Research studies have shown that POT could stimulate ALP activity in OB and upregulate bone morphogenetic protein-2 after POT intervention and also cause the upregulation of BMP-2 through Schnurri-2 and activated transcription factor 4 (ATF4) pathways, thus stimulating OB to differentiate into the mineralized phenotype (Tamma et al., 2009; Qian et al., 2015). In addition, Moghazy et al. (2020) have shown that intraperitoneal administration of POT has an anti-OP effect on rats. The mechanism is associated with the increasing number of osteocytes and OB, the decreasing levels of bone-specific alkaline phosphatase (bALP), osteocalcin, and tartrate-resistant acid phosphatase (TRAP), and the regulation of the expression of the OPG/RANKL pathway. Study has also explored how POT combined with OTR affected OB mineralization. It was discovered that when stimulated by POT, OTR could promote the translocation of OTR to the OB nucleus via continuous interaction with *β*-arrestins (Arrbs), the small GTPase Rab5, importin (Kpnb1), and transporter-1 (Tnpo1) and the differentiation and mineralization of OB (Di Benedetto et al., 2014). However, knockout of Arrb1, Arrb2, or Tnpo1 genes mediated by siRNA could eliminate expression of osterix (Sp7), Atf4, bone sialic acid protein, and osteocalcin that differentiated in OB induced by POT. It was vital to show that it did not affect the phosphatidylinositol three kinases/protein kinase B phosphorylation and the mitogen-activated protein kinase (MAPK) pathway.

Furthermore, POT and arginine vasopressin (Avp) have opposing effects on bone mass via high-affinity G protein-coupled receptors and so do their receptors. OTR is not indispensable for the role of Avp in inhibiting OB production and gene expression. However, when Avp1α knockout resulted in OTR deletion in cells, Avp-stimulated gene expression is inhibited, implying that Avp and POT may share receptors for the function of OB in regulating bone formation (Sun et al., 2016). Therefore, POT promotes the differentiation of OB into the mineralized phenotype, which may be the critical link to increasing bone mineral density and bone strength.

In summary, as a paracrine–autocrine regulator of bone formation, POT produced by OB promotes the translocation of OTR to the OB nucleus, increases the activity of ALP, controls the expression of OPG/RANKL and other related pathways, and upregulates bone mineralization and form factors, such as osteocalcin, BMP-2, and Runx2.

### 3.3 Osteoclasts

OC originate from the hematopoietic mononuclear-macrophage system, which are mainly responsible for decomposing and absorbing organic matter and minerals in the bone matrix, removing old damaged bones, and playing an essential role in bone development, growth, repair, and reconstruction (Muruganandan et al., 2020). OTR is highly expressed in OC (Assinder, 2022). In mature OB, POT induces the differentiation of OC by reducing the level of OPG and stimulating the expression of RANKL in OB (Qin et al., 2013). POT has a bi-directional regulatory effect on OC. On the one hand, it directly increases the formation of OC by activating nuclear factor kappa-B and MAPK signals, as well as indirectly increases the formation of OC by upregulating RANKL (Qin et al., 2013). On the other hand, it could reduce bone resorption by 40% through inducing intracytoplasmic Ca^2+^ release and nitric oxide synthesis after 48 h stimulated by POT in mature OC (Qin et al., 2013). In addition, the signal cascade induced by POT stimulates the release of intracytoplasmic Ca^2+^ and triggers the expression of extracellular regulated protein kinases in OB and OC. Studies have shown that the number of OC in the bone marrow of ovariectomized rats or mice decreased significantly, and bone mineral density increased markedly after POT treatment. The mechanism may be to restore the coupling of OB or OC *in vivo* and *in vitro* by increasing or decreasing the RANKL/OPG ratio (Moghazy et al., 2020).

In short, OC expresses POT and OTR, which regulates the ratio of OPG/RANKL in OB by triggering intracytoplasmic Ca^2+^ release and nitric oxide synthesis, which has both inhibitory and promoting effects on OC.

### 3.4 Osteocytes

Osteocyte is the most numerous and widely dispersed cell type in bones. It is made up of a small amount of OB that is embedded in the bone matrix and belongs to the terminal differentiation of OB (Mosialou et al., 2017). Osteocytes are distributed in the bone lacunae and surrounded by the bone matrix. Bone cells are connected with each other through linear pseudopods, and then the bone surface and OB are connected, which plays an important role in the network of bone lacunar and bone tubules (Wee et al., 2021). Studies have illustrated that osteocytes interact with RANKL secreted by OB, which influences OC production (Davis et al., 2017). In addition, further research has shown that bone cells and the OPG/RANKL ratio in the bone marrow of ovariectomized rats were significantly lower than those in normal rats. In contrast, after POT intervention, bALP, osteocalcin, and TRAP levels in the serum of ovariectomized rats decreased remarkably, while the OPG/RANKL ratio increased and osteocalcin and bone mineral density improved (Qian et al., 2015; Fernandes-Breitenbach et al., 2022). The OPG/RANKL signaling pathway is the classic bone coupling interaction pathway. The change in protein expression of OPG and RANKL determines bone resorption and formation direction.

In short, osteocytes, as terminal differentiations of OB, also express POT. POT increases the number of osteocytes and bone mineral density in ovariectomized rats by increasing the OPG/RANKL ratio and downregulating the bone resorption markers.

### 3.5 Chondrocytes

Chondrocytes are the only cartilage cells that are differentiated from bone marrow mesenchymal cells. It can produce and maintain cartilage matrix, including collagen and proteoglycan (Su et al., 2022). Normal cartilage development is important for bone formation in endochondral ossification. OP and osteoarthritis are caused in an abnormal microenvironment in the bone matrix (Roux et al., 2020). POT is expressed in chondrocytes, acts on subchondral bone, and stimulates cartilage formation by combining with OTR (Wu et al., 2017). Studies have shown a significant correlation between OA patients and low levels of POT (Ferrero et al., 2021). The expression of POT in chondrocytes of OA patients is reduced and responds dose-dependently to tumor necrosis factor (TNF-α) treatment. Simultaneously, POT may be beneficial to cartilage, subchondral bone, muscle, and inflammation (Ferrero et al., 2021). In addition, scholars have also found that POT reversed the gene and protein expression of MMP-1 and MMP-13 in chondrocytes through the JAK2/STAT1 pathway in a dose-dependent manner. In contrast, gene knockout of OTR eliminated the inhibitory effect of POT on MMP-1 and MMP-13 (Wu et al., 2017).

To summarize, chondrocytes express POT and promote cartilage production via binding to OTR. Furthermore, by regulating matrix metalloproteinases, POT prevents cartilage matrix destruction, whereas OTR inhibits the bone immune inflammatory reaction, which may be the key to improving bone microstructure.

### 3.6 Adipocytes

Adipocytes develop from the differentiation of BMSCs, similar to OB (Liu et al., 2021). Mesenchymal stem cells have the same ability to differentiate into adipocytes and OB under physiological conditions. However, under pathological conditions, when osteogenic or adipogenic regulation is dominant, the ability of mesenchymal stem cells to differentiate in the other direction usually is weakened (Muruganandan et al., 2020). Previously, it was thought that OB and bone marrow stromal cells were the main cells, which express RANKL/OPG in bone tissue and play an essential role in regulating OC differentiation (Wang et al., 2022). However, recent research has discovered that adipocytes express RANKL/OPG which also play an important role in influencing OC development and regulating bone remodeling (Matsuo et al., 2020). Indeed, intraperitoneal injection with OT negatively modulates adipogenesis while promoting osteogenesis (Camerino, 2009). The differentiation of BMSCs into adipocytes is the key to bone marrow obesity and OP in postmenopausal women (Rajapakse et al., 2021). It is demonstrated that POT inhibits adipocyte precursor differentiation and reverses osteopenia and bone marrow obesity in mice induced by ovariectomy, via regulating the signaling pathway of receptor activator between nuclear factor kappa-B and OPG axis (Beranger et al., 2014). POT could regulate the increase in ALP activity and the decrease in glyceraldehyde-3-phosphate dehydrogenase activity at different concentrations, through the culture of human pluripotent adipose-derived stem cells and BMSCs under the premise of any cell differentiation (Wang et al., 2022). This implied that POT stimulates OB differentiation while inhibiting adipocyte differentiation in a dose-dependent manner. Additionally, it was demonstrated that POT could control BMSC balance and coordination between OB and adipocytes *in vitro* (Wang et al., 2022).

In a word, adipocyte differentiation is intrinsically related to bone metabolism and affects the differentiation or function of bone tissue cells. POT inhibits adipocyte differentiation by regulating the OPG/RANKL signaling pathway and stimulating OB differentiation in a dose-dependent manner, which determines bone formation rather than fat formation.

## 4 OT/OTR feed-forward loop

Estrogen deficiency is a crucial factor that leads to a remarkable increase in osteocyte apoptosis in bone biopsy samples of premenopausal and postmenopausal women (Tomkinson et al., 1997). Mechanical and microenvironmental conditions for osteocytes will change with the progress in estrogen deficiency. The increased apoptosis would cause an excess of mineralization and then aggravate their paracrine response to OC (McNamara, 2021). The estrogen receptor is considered a positive factor in OB differentiation and bone formation and maintains bone formation through OB progenitor cells rather than mature OB (Jilka et al., 2014; McKay and Counts, 2020).

**FIGURE 1:**
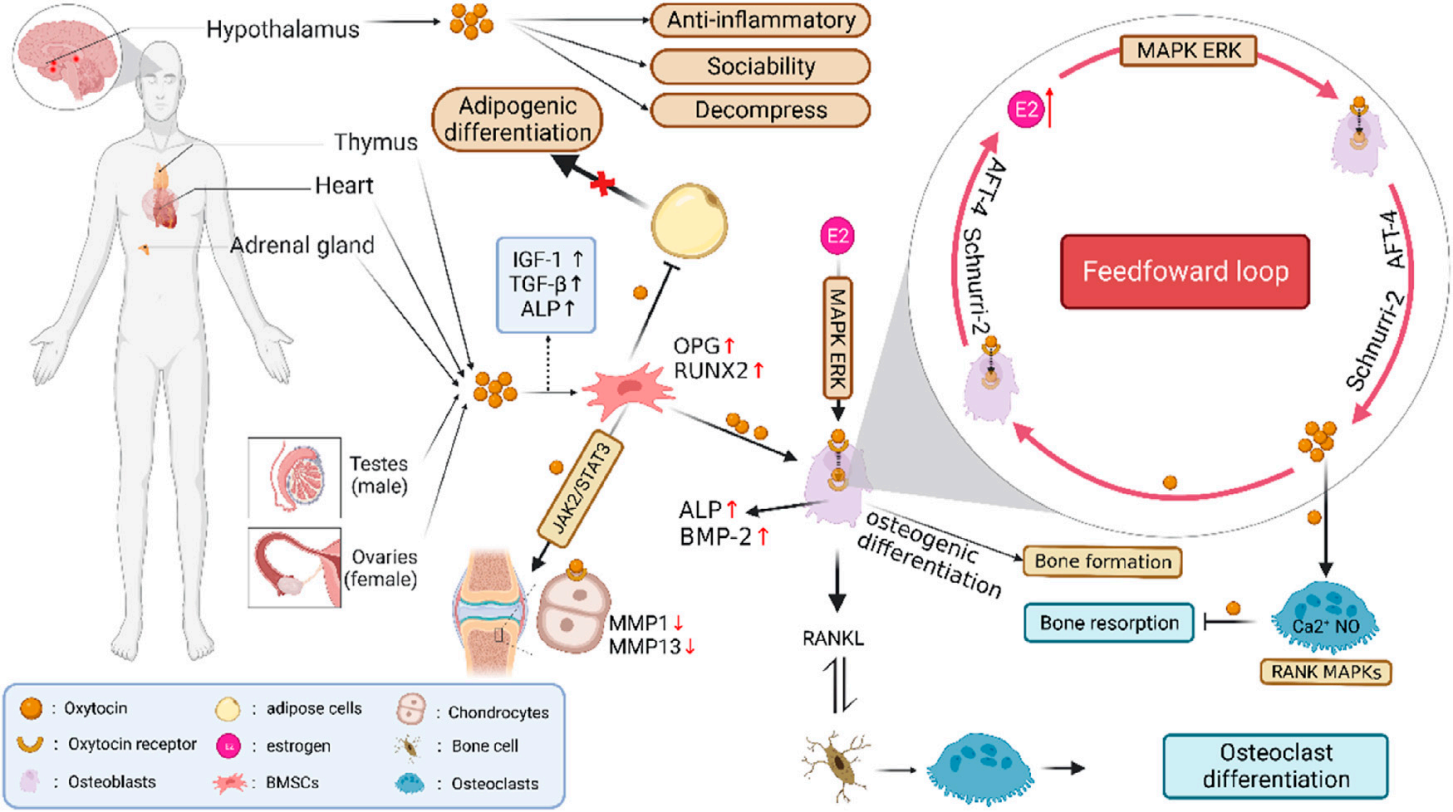
Feed-forward loop formed by POT/OTR and the metabolism in regulating bone cells. Note: The OT/OTR feed-forward loop is composed of OT, OTR, and estrogen. OT and estrogen are input regulators, while OTR is an output factor. After OB is affected by estrogen, it will produce OT reactively. When OT and its own receptor OTR combine with OB, it will further amplify the effect of estrogen and increase the production of OTR, thus forming the estrogen-mediated OT/OTR feed-forward loop. POT is produced in adipocytes, uterus, ovary, testis, and other tissues. OT and OTR are expressed in BMSCs, OB, OC, osteocytes, chondrocytes, and adipocytes and play a direct role in bone homeostasis. OT could promote the proliferation of BMSCs by regulating OPG/RANKL and MAPK/ERK signaling pathways, induce their differentiation and mineralization toward OB, increase the number of osteocytes, inhibit their differentiation toward osteoclasts and adipocytes, and improve the level of bone mineral density. In addition, OT regulates the JAK2/STAT3 pathway in a dose-dependent manner by combining with OTR, inhibiting the bone immune inflammatory reaction, and improving bone microstructure.

In previous studies, researchers have paid more attention to some functions of the OT system, such as social grooming and sexual behaviors that support the formation and maintenance of social bonds through a positive feedback loop (Nagasawa et al., 2015; Brooks et al., 2022). The OT/OTR feed-forward loop is a new cyclic linear mode sequence for regulating bone metabolism, which is composed of two input regulators (OT and estrogen) and one output factor (OTR) (McKay and Counts, 2020) (Figure 1). The anabolism of estrogen in bone occurs partially at least through the autocrine OT/OTR feed-forward loop. After being affected by estrogen, OB usually produces OT reactively. After OT and OTR being combined with OB, it would further amplify the effect of estrogen, increase the production of OT, and then form the OT/OTR feed-forward loop mediated by estrogen (Fallahnezhad et al., 2020). OB that expressed OTR highly could synthesize OT. OT and OTR genes are transcribed under the control of estrogen. The local synthesis of OT in OB is mediated by estrogen. It was shown that OB could synthesize and secrete OT after estradiol intervention within 2 h, and estrogen could regulate the activity of bone cells via OT. Indeed, the hypodermic injection of OT could prevent and reverse bone loss in ovariectomized mice by improving the bone microarchitecture, including trabecular number, trabecular spacing and total bone volume, and enhancing biomechanical strength (Qiu et al., 2019; Breuil et al., 2021). However, OTR is required for estrogen synthesis and metabolism in the OB, implying that OT is the synthetic metabolic medium of estrogen acting on bones (Colaianni et al., 2011; McCormack et al., 2020).

## 5 Discussion and conclusion

With the pleiotropic actions on body composition, OT could elicit a plethora of biological responses via OTR in both the peripheral and central nervous system, including maternal behaviors, cancer, social bonding, milk ejection, uterus contraction, menopause, cognitive functions, cardiovascular diseases, and stress. Several studies have shown that social psychological behaviors, such as attachment, human sociability, anger, and fear, were influenced by environment interaction mediated by epigenetic DNA modification (Puglia et al., 2015; Haas et al., 2016). As a typical member of the G protein-coupled receptor family, OT and OTR represent the intriguing target for cancer therapy, including craniopharyngioma, breast cancer, and ovarian cancer. Research has showed that, due to its functions in the immune system, dysregulation of OT/OTR in breast tumor tissues could also be linked with the immune escape mechanism of the cancer cells (Li et al., 2016; Liu et al., 2020).

PMOP, osteoporosis, and osteoporosis fracture have become a worldwide public health problem (Jones et al., 2021). Teriparatide, bisphosphonates, estrogen, calcitriol, and fluoride are commonly applied for treating and preventing PMOP, osteoporosis, and osteoporosis fracture (Compston et al., 2017). Recent data have demonstrated that the anabolic effect of OT on bone and the level of plasma OT represent a novel diagnostic marker for osteoporosis (Moerkerke et al., 2021). Research has shown that the action of OT differs with OTR on bone metabolism according to gender (Masi et al., 2021). OT levels were higher in premenopausal than postmenopausal women. A set of clinical survey data on 1,097 postmenopausal women has shown that the correlation between the OT serum level and BMD remains significant at the hip in women with unmeasurable estradiol (Breuil et al., 2014). However, currently, there was no high-quality research evidence of human data available on the beneficial effects of OT as a treatment and more generally on all menopause-associated diseases.

Interestingly, the experiment of cold stress challenge showed an obvious decrease in the expression of OXT gene in BAT after 6 h but an increase in the bone after 5 days, while OXTR gene is upregulated in brain consistently, which supports the concept of a coordinated axis mediated by OXT between bone and energy (Camerino et al., 2019). It suggests that OT plays a main role in this tissue in response to cold stress challenge.

These data reinforce the fact that the direct effect of OT on the bone is mainly related to POT rather than COT. OB synthesizes POT under the stimulation of estrogen, which becomes a paracrine–autocrine regulator of bone formation and highly expresses OTR. OT and OTR are expressed in BMSCs, OB, OC, osteocytes, chondrocytes, and adipocytes. OT, OTR, and estrogen form a feed-forward loop that directly regulates bone metabolism. To increase the activity of BMSCs and promote their differentiation into OB rather than adipocytes, OT can regulate the OPG/RANKL signaling pathway, upregulate osteocalcin, OPG, RUNX2, and other BMPs, and downregulate the levels of bALP, osteocalcin, and TRAP bone resorption markers. Then, OT induces OB differentiation and mineralization by promoting OTR translocation into the nucleus. OT regulates the OPG/RANKL ratio in OB by inducing the release of intracytoplasmic Ca^2+^ and the synthesis of nitric oxide, leading to a bidirectional regulatory effect on OC. In addition, OT can also increase the activity of osteocytes and chondrocytes that play a vital role in increasing bone mass and improving bone microstructure. Therefore, more in-depth research studies should focus on improving the harmfulness from osteoporosis and osteoporosis fractures.

OT and OTR are critical in maintaining the balance between bone formation and bone resorption. The potential regulatory effects of POT and OTR on bone metabolism require further investigations. They are beneficial to the potential of bone formation and provide scientific ideas and theoretical basis for the application of OT and OT analogues in the prevention and treatment of osteoporosis and the development of new drugs.

However, this research has several limitations. First, although there are many animal experimental studies on the intervention of OT and OTR in the bone, high-quality clinical studies and drug efficacy evidence in humans are still insufficient. Second, further analysis is needed on the key targets of OT and OTR in anti-osteoporosis. Third, this research has only reviewed the mechanism of action of bone metabolism-related cells, and the molecular mechanism of OT and OTR to improve bone microstructure still needs to be further explored.
